# Non-toxigenic *Corynebacterium diphtheriae* infective endocarditis with embolic events: a case report

**DOI:** 10.1186/s12879-020-05652-w

**Published:** 2020-12-01

**Authors:** Antonio de Santis, Rinaldo Focaccia Siciliano, Roney Orismar Sampaio, Masahiko Akamine, Elinthon T. Veronese, Francisco Monteiro de Almeida Magalhaes, Maria Rita Elmor Araújo, Flavia Rossi, Marcelo M. C. Magri, Ana Catharina Nastri, Tarso A. D. Accorsi, Vitor E. E. Rosa, David Provenzale Titinger, Guilherme S. Spina, Flavio Tarasoutchi

**Affiliations:** 1 Heart Valve Unit, Heart Institute (InCor), University of São Paulo Medical School, Av. Dr. Eneas de Carvalho Aguiar, 44, São Paulo, SP 05403-000 Brazil; 2 Hospital Israelita Albert Einstein, Sao Paulo, Brazil; 3 Infection Control Team, Heart Institute (InCor), University of Sao Paulo Medical School, Sao Paulo, Brazil; 4 General Surgery Department, Heart Institute (InCor), University of Sao Paulo Medical School, Sao Paulo, Brazil; 5 Cardiac Surgery Department, Heart Institute (InCor), University of Sao Paulo Medical School, Sao Paulo, Brazil; 6 Clinical Microbiology Laboratory, Hospital das Clinicas, University of Sao Paulo Medical School, Sao Paulo, Brazil; 7 Department of Infectious Diseases, Hospital das Clinicas, University of Sao Paulo Medical School, Sao Paulo, Brazil

**Keywords:** Infective endocarditis, Cardiac surgery, Embolism, Abscess

## Abstract

**Background:**

*Corynebacterium diphtheriae* (*C. diphtheriae*) infections, usually related to upper airways involvement, could be highly invasive. Especially in developing countries, non-toxigenic *C. diphtheriae* strains are now emerging as cause of invasive disease like endocarditis. The present case stands out for reinforcing the high virulence of this pathogen, demonstrated by the multiple systemic embolism and severe valve deterioration. It also emphasizes the importance of a coordinated interdisciplinary work to address all these challenges related to infectious endocarditis.

**Case presentation:**

A 21-year-old male cocaine drug abuser presented to the emergency department with a 1-week history of fever, asthenia and dyspnea. His physical examination revealed a mitral systolic murmur, signs of acute arterial occlusion of the left lower limb, severe arterial hypotension and acute respiratory failure, with need of vasoactive drugs, orotracheal intubation/mechanical ventilation, empiric antimicrobial therapy and emergent endovascular treatment. The clinical suspicion of acute infective endocarditis was confirmed by transesophageal echocardiography, demonstrating a large vegetation on the mitral valve associated with severe valvular regurgitation. Abdominal ultrasound was normal with no hepatic, renal, or spleen abscess. Serial blood cultures and thrombus culture, obtained in the vascular procedure, identified non-toxigenic *C. diphtheriae*, with antibiotic therapy adjustment to monotherapy with ampicillin. Since the patient had a severe septic shock with sustained fever, despite antimicrobial therapy, urgent cardiac surgical intervention was planned. Anatomical findings were compatible with an aggressive endocarditis, requiring mitral valve replacement for a biological prosthesis. During the postoperative period, despite an initial clinical recovery and successfully weaning from mechanical ventilation, the patient presented with a recrudescent daily fever. Computed tomography of the abdomen revealed a hypoattenuating and extensive splenic lesion suggestive of abscess. After sonographically guided bridging percutaneous catheter drainage, surgical splenectomy was performed. Despite left limb revascularization, a forefoot amputation was required due to gangrene. The patient had a good clinical recovery, fulfilling 4-weeks of antimicrobial treatment.

**Conclusion:**

Despite the effectiveness of toxoid-based vaccines, recent global outbreaks of invasive *C. diphtheriae* infectious related to non-toxigenic strains have been described. These infectious could be highly invasive as demonstrated in this case. Interdisciplinary work with an institutional “endocarditis team” is essential to achieve favorable clinical outcomes in such defiant scenarios.

## Background

Diphtheria, a disease characterized by the presence of inflammatory pseudomembranes on the tonsils and upper respiratory tract, was first described by Loeffler in 1884, establishing the causal relationship between the disease and its agent: *Corynebacterium diphtheriae (C. diphtheriae)* [1]. Actually, subsequent microbiological evidence demonstrated that the infection pathogenicity was related to a bacterial toxin, enabling the development of an effective anti-toxin vaccine and epidemiological control of the disease. However, especially in developing countries, non-toxigenic *C. diphtheriae* strains are now emerging as cause of invasive disease like endocarditis, septic arthritis and osteomyelitis [1, 2]. In fact, there is an increase in case reports of severe infection by non-toxigenic *C. diphtheriae* over the past few decades [3–6]. Non-toxigenic *C. diphtheriae* endocarditis has been related to the presence of valve prosthesis, use of injectable drugs and underlying cardiac disease. Muttaiyah et al., in a 10 patients’ case series of *C. diphtheriae* endocarditis, reported a favorable prognosis with a low complication rate and no death record [6].

We present a non-toxigenic *C. diphtheriae* endocarditis case report with multiple embolic events and severe valve damage, reinforcing the virulence potential of this organism. Moreover, this case stands out for emphasizing the importance of an institutional “endocarditis team” to address all the challenges related to this disease.

## Case presentation

**Fig. 1:**
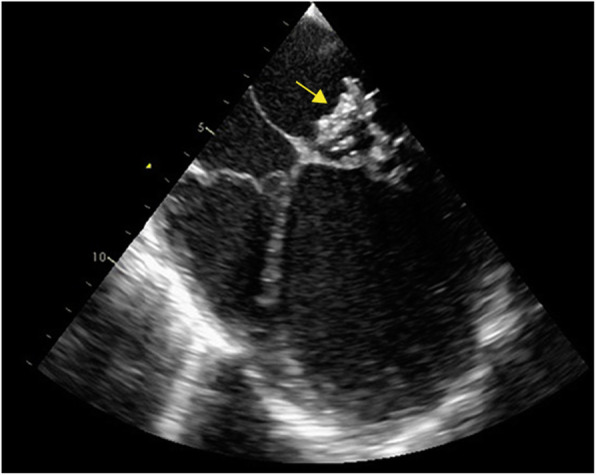
Large mitral vegetation (yellow arrow) in the transesophageal echocardiography

A 21-year-old male cocaine drug abuser presented to the emergency department with a 1-week history of fever, asthenia and dyspnea. There were no other respiratory symptoms including sore throat or cough. Despite drug addiction, his past medical history was unremarkable, with no evidence of underlying cardiac disease or known immunosuppression condition. He also denied intravenous drug use. Additionally, he presented an updated vaccination record, including the diphtheria-pertussis-tetanus (DPT) vaccine. On examination, he was febrile at 38 °C, hypoxemic (SpO2 85%), tachypneic (28 breaths per minute), tachycardic at 120 bpm and with severe arterial hypotension (80/40 mmHg). On auscultation, there was a grade III mitral systolic murmur and diffuse crackles in all lung areas. Physical examination also revealed signs of acute arterial occlusion of the left lower limb. Blood tests revealed a white cell count of 27.050/mm^3^ (normal value 4.0–11.0), neutrophils of 22.452/mm^3^ (normal value 2.0–7.5), hemoglobin of 8.2 g/dL (normal value 13.5–17.5), creatinine of 2.99 mg/dL (normal value 0.7–1.3), arterial lactate of 50 mg/dL (normal value 4–14) and C-reactive protein was 320 mg/L (normal value 0.0–5.0). HIV testing, through enzyme-linked immunosorbent assay, was negative. The 12-lead electrocardiogram showed sinus tachycardia. Chest x-ray findings were compatible with pulmonary congestion (Kerley’s B lines and bilateral reticular opacities). Vascular ultrasound of the left lower limb showed anterior tibial artery occlusion at the proximal segment. The severity of the clinical presentation on admission, imposed prompt orotracheal intubation and mechanical ventilation, vasoactive drugs, empiric antimicrobial therapy and emergency endovascular treatment with mechanical thrombectomy of the left anterior tibial artery for clot retrieval. The clinical suspicion of acute infective endocarditis was confirmed by transesophageal echocardiography, demonstrating a large mitral vegetation (18 × 15 mm) associated with severe mitral regurgitation caused by leaflets perforations (Fig. 1). Abdominal ultrasound was normal with no hepatic, renal, or spleen abscess. Serial blood cultures and thrombus culture, obtained in the vascular procedure, identified non-toxigenic *C. diphtheriae*, with antibiotic therapy adjustment to monotherapy with ampicillin. Pathogen identification was achieved through automated blood culture - BD BACTEC FX (Becton Dickinson Diagnostics, Sparks, USA), manual seeding on 5% sheep blood agar (abundant bacterial growth in less than 24 h) and confirmed by Matrix Assisted Laser Desorption Ionization-Time of Flight Mass Spectrometry (MALDI-TOF MS). Polymerase chain reaction assay, using the colonies from the agar plate, did not detect the tox gene, characterizing a non-toxigenic strain. Antibiotic susceptibility testing was not performed due to the absence of standardized procedures for *C. diphtheriae* evaluation. Obtained cultures were exclusive positive for *C. diphtheriae*.

**Fig. 2:**
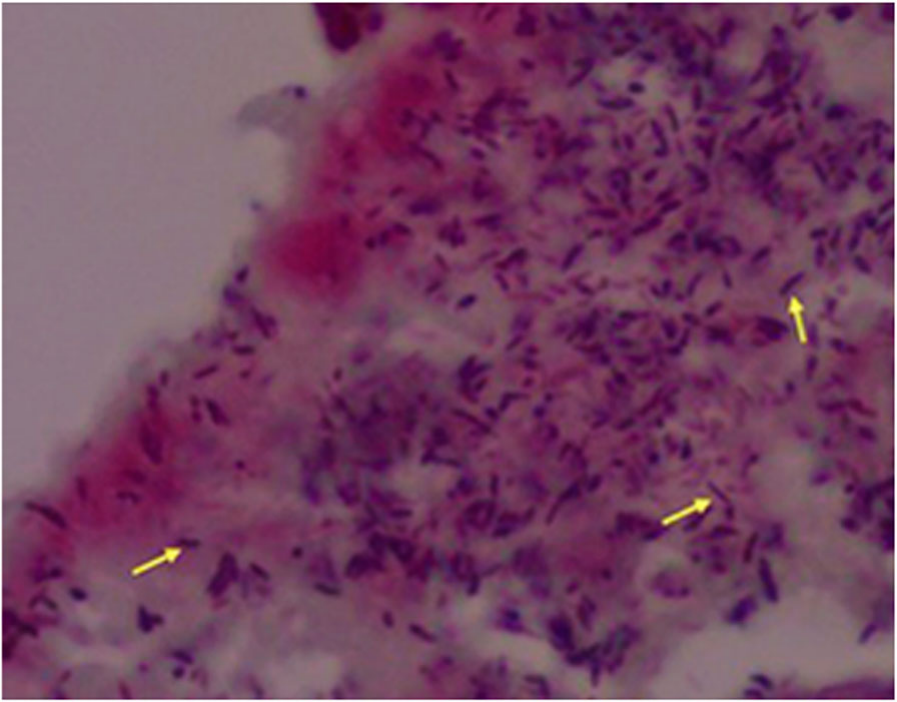
Histopathology of the mitral valve with Gram-positive bacilli (yellow arrows)

**Fig. 3:**
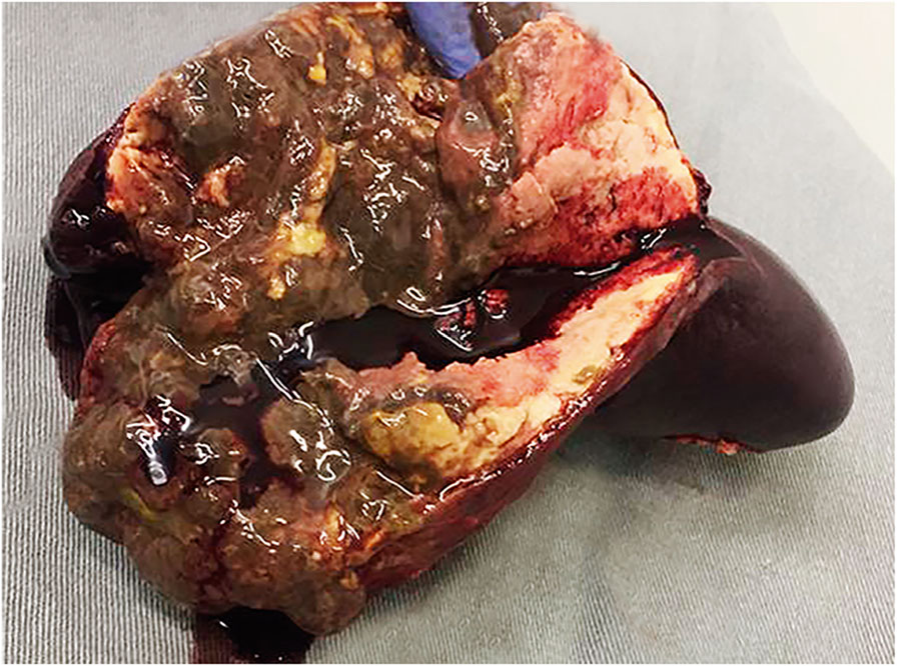
Macroscopic image of the excised spleen with a large abscess

**Fig. 4:**
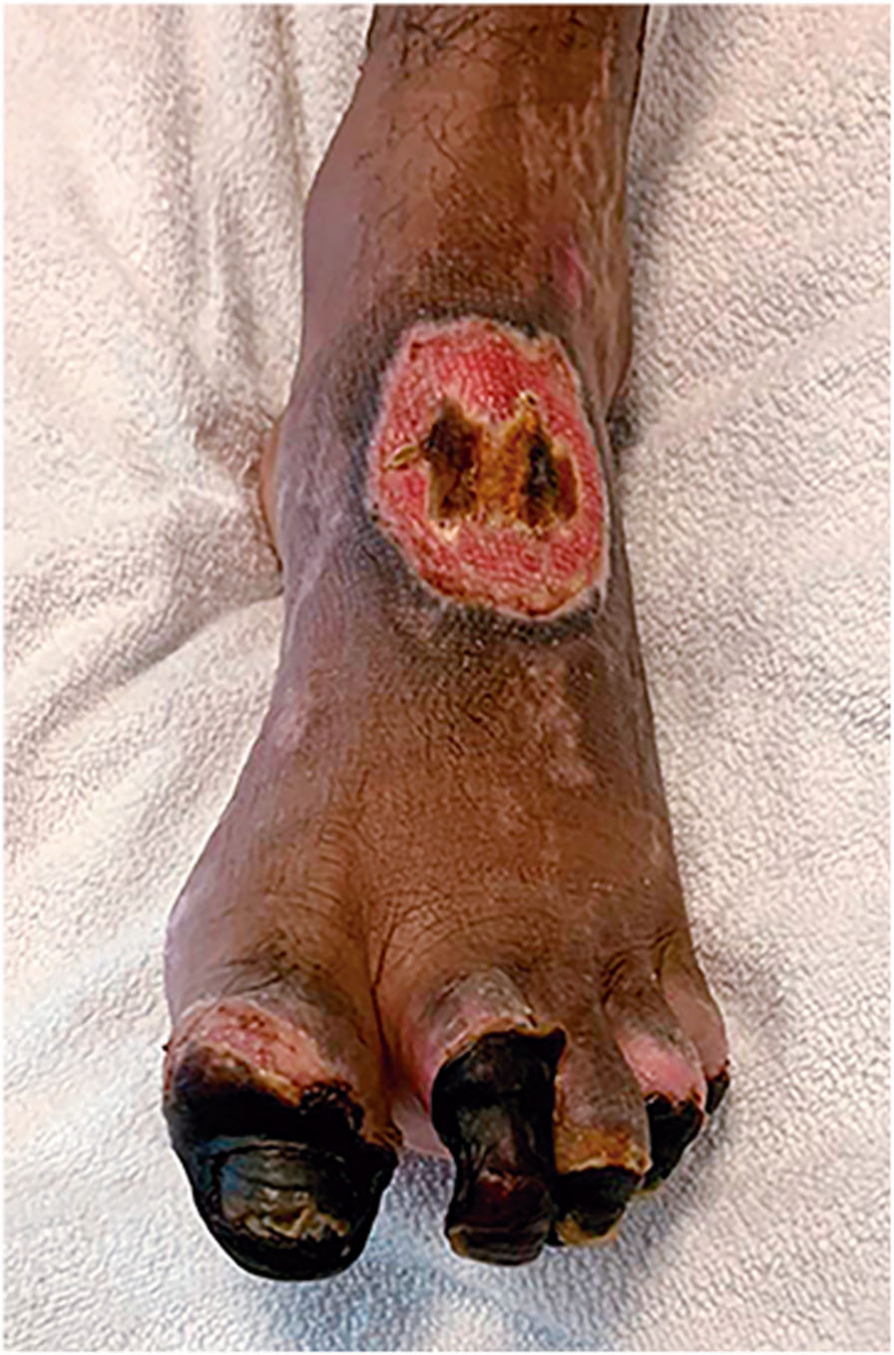
Forefoot gangrene related to septic vascular embolism

The case met two majors’ conditions of the Duke’s modified diagnostic criteria for infective endocarditis: echocardiogram evidence of vegetations and positive blood culture [7]. Since the patient had a severe septic shock with sustained fever, despite guided antimicrobial therapy, urgent cardiac surgical intervention was planned after 4-days of hospitalization. The intraoperative anatomical findings were compatible with an aggressive endocarditis: numerous vegetations on the mitral valve and perforated leaflets, requiring mitral valve replacement for a biological prosthesis. The histopathological analysis confirmed a bacterial infective endocarditis, demonstrating Gram-positive bacilli (Fig. 2) with positive valve culture for *C. diphtheriae*. Postoperative transthoracic echocardiography exhibited a preserved left ventricular ejection fraction and proper functioning of the mitral bioprosthesis. During the postoperative period, regardless of an initial clinical recovery and successfully weaning from mechanical ventilation, the patient presented with a recrudescent daily fever (39 °C) and lower left back pain. Computed tomography (CT) of the abdomen revealed a hypoattenuating and extensive splenic lesion suggestive of abscess. Sonographically guided bridging percutaneous catheter drainage was performed, with removal of 100 mL of purulent secretion. Even though, daily fever still remained, requiring surgical splenectomy 5 days after. The macroscopic analysis of the spleen confirmed the presence of a large abscess (Fig. 3). Cultures obtained from splenic samples were also positive for *C. diphtheriae*. The patient had a good post-procedure clinical recovery and fever resolution after 2 days. However, despite early left limb revascularization, a forefoot amputation was required due to distal gangrene (Fig. 4). Despite all these challenging complications, the patient had a good clinical recovery and received hospital discharge after fulfilling 4-weeks of antimicrobial treatment. Table 1 presents a timeline with the main events and respective medical interventions.

**Table 1:** Timeline with the main clinical events and interventions

Time	Day 0	Day 4	Day 10	Day 15	Day 23	Day 32
Events	• Hospitalization • Mitral endocarditis diagnosis • Acute left tibial artery occlusion	• Septic shock • Sustained fever • Severe mitral regurgitation	• Daily fever • Splenic abscess diagnosis	• Recurrent fever • Persistence of splenic abscess	• Distal left lower limb gangrene	• Clinical recovery
Interventions	• Orotracheal intubation and mechanical ventilation • Antimicrobial therapy • Vascular thrombectomy	• Mitral valve replacement surgery	• Percutaneous splenic drainage	• Surgical splenectomy	• Left forefoot amputation	• Hospital discharge

## Discussion and conclusions

The global immunization against *C. diphtheriae*, based on an anti-toxin vaccine, has been fundamental in controlling the morbidity and mortality of diphtheria, especially in children. However, the vaccine does not prevent infections by non-toxigenic strains which can be highly aggressive. Due to its rarity, the medical literature presents non-toxigenic *C. diphtheriae* endocarditis mainly through case reports and reviews, with scarce descriptions of early embolic phenomena as acute ischemic strokes, vascular pseudoaneurysms and septic arthritis [3–6].

Our patient is young, with no previously known clinical comorbidities or underlying cardiac disease, but with early exposure to illicit drugs. He reported intranasal cocaine use since adolescence. Despite the well-known association of endocarditis with injectable drugs abuse, the use of inhaled drugs as cocaine could also trigger periods of transient bacteremia due to direct injury to the nasal mucosa [8]. Additionally, some patients with upper airways colonization by non-toxigenic C. diphtheriae could be more prone to transient bacteremia by this agent in situations involving local barrier break. This could be one of the possible etiopathogenic mechanisms involved in this case.

The present case stands out for the clinical aggressiveness of the disease. Social vulnerability related to drug addiction limited the patient’s access to medical assistance in proper time. He was admitted to the hospital with acutely installed respiratory failure and septic shock, requiring immediate invasive clinical intervention. A previous case series study in a young population composed by 10 patients reported favorable outcomes in non-toxigenic *C. diphtheriae* endocarditis: low need for surgical intervention (30%), no hospital mortality record and just one case of septic embolism [6]. Conversely, in the present case, the agent’s virulence is noticed right on hospital admission, reinforcing the need for an individualized approach for each case. Additionally, the case complexity required an intense interdisciplinary work with the participation of several professionals (clinical cardiologist, microbiologists, surgeons and interventional radiologists). This “endocarditis team” is essential to ensure favorable outcomes in such challenging scenarios [9].

Regarding vascular complications, arterial embolism of valve vegetations are frequent, diverse and often impose the need for immediate intervention therapy. Arterial embolisms to the lower limbs are reported in 20 to 30% of cases [10]. The peak incidence of these phenomena is usually in the first 2 weeks of antimicrobial treatment, being influenced by the size and mobility of vegetation [11]. In our case, the transesophageal echocardiography images demonstrated the presence of a very large vegetation (18 mm) that certainly was determinant for the multiple related embolic complications.

Even with antimicrobial therapy, our patient remained hemodynamically unstable, with daily fever and persistently elevated inflammatory blood tests, indicating a failure of etiological treatment with need of cardiac valve replacement surgery. The evidence for antimicrobial therapy in non-toxigenic *C. diphtheriae* infections is sparse and limited. The lack of standardized procedures for antibiotic testing is a limiting factor, although the community strains of non-toxigenic *C. diphtheriae* are usually susceptible to penicillin and macrolides, with rare cases of antibiotic resistance [12].

Splenic abscess in left heart infective endocarditis is a very rare condition (around 5%), usually related to direct seeding of the spleen by an infected embolus [13]. Clinically, persistent fever, maintenance of bacteremia and low back pain are suggestive of splenic abscess. In this case, the CT-scan images were compatible with the diagnosis, with the typical hypoattenuating aspect of the lesion. Five-days after percutaneous drainage, splenectomy was performed with definitive resolution of the postoperative fever. It is worth mentioning that splenectomy should be performed before valve-replacement surgery as the abscess manipulation during the surgical procedure may lead to infection of the valve prosthesis as a result of the bacteremia.

From an educational point of view, the present case leads to some important conclusions: (1) despite the impact of global immunization for Diphtheria, occurrence of invasive infection related to non-toxigenic *C. diphtheriae* strains is possible, especially in populations with greater vulnerability (immunocompromised, drug addicts), (2) skilled institutional groups (“endocarditis teams”) are essential for the proper management of this condition. This coordinated interdisciplinary action allows accurate diagnosis, facilitates the decision-making process and reduces the mortality related to infectious endocarditis.

## Data Availability

All data generated during this study are included in this published article.

## Abbreviations

*C. diphtheriae*: *Corynebacterium diphtheriae*
CT: Computed tomography
DPT: Diphtheria-pertussis-tetanus vaccine
MALDI-TOF MS: Matrix Assisted Laser Desorption Ionization-Time of Flight Mass Spectrometry

## References

[1] Pachirat O, Kaewkes D, Pussadhamma B, Watt G (2018). Corynebacterium diphtheriae native aortic valve endocarditis in a patient with prosthetic mitral valve: a rare presentation. Cardiol Res.

[2] Patris V, Argiriou O, Konstantinou C, Lama N, Georgiou H, Katsanevakis E, Argiriou M, Charitos C (2014). Corynebacterium diphtheriae endocarditis with multifocal septic emboli: can prompt diagnosis help avoid surgery?. Am J Case Rep.

[3] El-Hazmi MM (2015). Late-onset prosthetic valve endocarditis caused by nontoxigenic Corynebacterium diphtheriae. J Infect Dev Ctries.

[4] Mishra B, Dignan RJ, Hughes CF, Hendel N (2005). Corynebacterium diphtheriae endocarditis--surgery for some but not all!. Asian Cardiovasc Thorac Ann.

[5] Ng J, Downton T, Davidson N, Marangou J (2019). Corynebacterium diphtheriae-infective endocarditis in a patient with an atrial septal defect closure device. BMJ Case Rep.

[6] Muttaiyah S, Best EJ, Freeman JT, Taylor SL, Morris AJ, Roberts SA (2011). Corynebacterium diphtheriae endocarditis: a case series and review of the treatment approach. Int J Infect Dis.

[7] Li JS, Sexton DJ, Mick N, Nettles R, Fowler VG, Ryan T, Bashore T, Corey GR (2000). Proposed modifications to the Duke criteria for the diagnosis of infective endocarditis. Clin Infect Dis.

[8] Chambers HF, Morris DL, Täuber MG, Modin G (1987). Cocaine use and the risk for endocarditis in intravenous drug users. Ann Intern Med.

[9] Davierwala PM, Marin-Cuartas M, Misfeld M, Borger MA (2019). The value of an "endocarditis team". Ann Cardiothorac Surg.

[10] Pessinaba S, Kane A, Ndiaye MB, Mbaye A, Bodian M, Dia MM, Sarr SA, Diao M, Sarr M, Kane A, Ba SA (2012). Vascular complications of infective endocarditis. Med Mal Infect.

[11] Thuny F, Di Salvo G, Belliard O, Avierinos JF, Pergola V, Rosenberg V, Casalta JP, Gouvernet J, Derumeaux G, Iarussi D, Ambrosi P, Calabró R, Riberi A, Collart F, Metras D, Lepidi H, Raoult D, Harle JR, Weiller PJ, Cohen A, Habib G (2005). Risk of embolism and death in infective endocarditis: prognostic value of echocardiography: a prospective multicenter study. Circulation..

[12] Zasada AA, Baczewska-Rej M, Wardak S (2010). An increase in non-toxigenic Corynebacterium diphtheriae infections in Poland--molecular epidemiology and antimicrobial susceptibility of strains isolated from past outbreaks and those currently circulating in Poland. Int J Infect Dis.

[13] Bayer AS, Bolger AF, Taubert KA, Wilson W, Steckelberg J, Karchmer AW, Levison M, Chambers HF, Dajani AS, Gewitz MH, Newburger JW, Gerber MA, Shulman ST, Pallasch TJ, Gage TW, Ferrieri P (1998). Diagnosis and management of infective endocarditis and its complications. Circulation.

